# Interpregnancy Interval After Clinical Pregnancy Loss and Outcomes of the Next Frozen Embryo Transfer

**DOI:** 10.1001/jamanetworkopen.2023.40709

**Published:** 2023-10-31

**Authors:** Ze Wang, Yueru Meng, Xue Shang, Lu Suo, Dingying Zhao, Xinwei Han, Min Yang, Mengfei Yin, Haozhe Miao, Yixuan Wang, Huiming Yang, Yunhai Yu, Daimin Wei, Zi-Jiang Chen

**Affiliations:** 1 Center for Reproductive Medicine, Shandong University, Jinan, China; 2 Department of Obstetrics and Gynecology, Second Hospital of Shandong University, Jinan, China; 3 Medical Integration and Practice Center, Shandong University, Jinan, China

## Abstract

**Importance:**

The optimal interpregnancy interval (IPI) after a clinical pregnancy loss (CPL) remains controversial. Few studies have addressed the role of the IPI after a preceding CPL during in vitro fertilization (IVF) treatment.

**Objective:**

To evaluate the association between different IPI lengths after a preceding CPL and pregnancy outcomes of the next frozen embryo transfer (FET).

**Design, Setting, and Participants:**

This retrospective cohort study was conducted using data from the Center for Reproductive Medicine of Shandong University in China. The study included women who underwent frozen-thawed blastocyst transfer between July 1, 2017, and June 30, 2022, within 1 year after a preceding CPL during IVF treatment. Follow-up for pregnancy outcomes was completed for all participants on March 31, 2023. Data analysis was performed from April to May 2023.

**Exposures:**

Interpregnancy interval length was classified as less than 3 months, 3 to less than 6 months, or 6 to 12 months.

**Main Outcomes and Measures:**

Outcomes included live birth, conception, clinical pregnancy, pregnancy loss, preterm birth, small or large for gestational age, and low birth weight. Multivariable logistic regression analysis was conducted to evaluate the association between IPI and pregnancy outcomes by adjusted odds ratios (AORs).

**Results:**

This study included 2433 women (mean [SD] age, 31.8 [4.6] years) who received IVF treatment. There were 338 women (13.9%) with an IPI of less than 3 months, 1347 (55.4%) with an IPI of 3 to less than 6 months, and 748 (30.7%) with an IPI of 6 to 12 months. The median (IQR) IPI lengths for the 3 groups were 77 (65-85), 128 (109-152), and 234 (202-288) days, respectively. Compared with an IPI of 6 to 12 months, shorter IPIs (<3 and 3 to <6 months) were associated with decreased odds of clinical pregnancy (AOR, 0.70 [95% CI, 0.53-0.92] and 0.79 [0.65-0.95]), live birth (AOR, 0.64 [95% CI, 0.48-0.85] and 0.74 [0.61-0.90]), and healthy live birth (AOR, 0.63 [95% CI, 0.46-0.87] and 0.79 [0.64-0.98]). Compared with women with an IPI of 6 to 12 months, women with shorter IPIs (<3 and 3 to <6 months) had a higher risk of total pregnancy loss (AOR, 1.87 [95% CI, 1.31-2.67] and 1.29 [1.00-1.66], respectively).

**Conclusions and Relevance:**

The results of this study suggest that delaying the next FET for at least 6 months after a preceding CPL was associated with beneficial pregnancy outcomes, considering that a decreased likelihood of achieving clinical pregnancy and live birth was observed among women with shorter IPIs. Further prospective studies are needed to confirm these findings.

## Key Points

**Question:**

What is the optimal interpregnancy interval (IPI) between a preceding clinical pregnancy loss and subsequent frozen embryo transfer in women undergoing in vitro fertilization (IVF) treatment?

**Findings:**

In this cohort study of 2433 women who received IVF treatment, a lower likelihood of achieving live birth was observed among women with a shorter IPI (<3 or 3 to <6 months) compared with those with an IPI of 6 to 12 months.

**Meaning:**

These results suggest that delaying frozen embryo transfer for at least 6 months after a preceding clinical pregnancy loss was associated with beneficial pregnancy outcomes.

## Teaser

This cohort study evaluates the association between interpregnancy interval length after a preceding clinical pregnancy loss and pregnancy outcomes of the subsequent frozen embryo transfer.

## Introduction

The time interval between the end of a pregnancy and the start of a subsequent pregnancy, known as the interpregnancy interval (IPI), may affect the outcomes of the index pregnancy. There is convincing evidence that a short IPI after a live birth (eg, <18 months) is correlated with increased risk of preterm birth, low birth weight, and small for gestational age (SGA) in the next pregnancy.^1,2^ Proposed mechanisms included maternal nutritional depletion, folate depletion, and unrecovered vascular remodeling of the uterus in women with short IPIs.^3,4^

The waiting time before attempting the next conception is usually shorter among women with a preceding conception that resulted in a clinical pregnancy loss (CPL) than those with a live birth. Among women undergoing in vitro fertilization (IVF), when to proceed with the next cycle of embryo transfer after a CPL that occurred during the preceding cycle of embryo transfer is one of the important clinical decisions. However, the optimal IPI after a CPL remains controversial.^5^ An early large-scale study in Latin America reported increased risks of preterm birth and low birth weight when the IPI was less than 6 months.^6^ A meta-analysis revealed that compared with an IPI of 6 months or longer, an IPI of less than 6 months after miscarriage was not associated with increased risk of adverse outcomes but was associated with lower risk of further miscarriage and preterm birth in the next pregnancy.^7^ Moreover, a large cohort study in Norway found no evidence of elevated risk of adverse pregnancy outcomes among women who conceived within 3 months after a spontaneous or induced abortion.^8^ Notably, most of these studies were conducted in naturally conceived pregnancies. Both the IPI and outcomes of the next pregnancy may be affected by the fecundity of participants in naturally conceiving populations. Whether these results could be generalized to women undergoing IVF warrants further study.

Interpregnancy interval length in women undergoing IVF is largely determined by the starting time of the next cycle of embryo transfer. Women who conceive by IVF treatment generally have an increased risk of adverse pregnancy outcomes compared with those who conceive naturally.^9^ Moreover, women who have experienced a CPL during IVF treatment are more prone to psychological distress. Nevertheless, few studies have addressed the optimal IPI after a preceding CPL in IVF populations.^10^ In this large-sample retrospective cohort study, we investigated the association between different IPI lengths after a preceding CPL and pregnancy outcomes of the next embryo transfer.

## Methods

### Study Population

This cohort study was approved by the Ethics Committee of the Center for Reproductive Medicine of Shandong University, and a waiver of informed consent was granted because this study was a retrospective analysis of deidentified data. We followed the Strengthening the Reporting of Observational Studies in Epidemiology (STROBE) reporting guideline.

Data were retrieved from the clinical database of the Center for Reproductive Medicine of Shandong University. This study included women who underwent frozen-thawed blastocyst transfer between July 1, 2017, and June 30, 2022, within 1 year after a CPL occurring during a preceding embryo transfer that was derived from the same ovarian stimulation cycle. Follow-up for pregnancy outcomes was completed for all participants on March 31, 2023. The exclusion criteria were as follows: (1) history of recurrent pregnancy loss (defined as ≥2 consecutive spontaneous abortions, including biochemical pregnancy loss); (2) history of recurrent implantation failure (defined as the failure to achieve a clinical pregnancy after ≥3 cycles of embryo transfer); (3) diagnosis of uterine abnormalities, including uterine congenital abnormalities as well as untreated submucosal myoma and endometrial polyp, and history of intrauterine adhesion; (4) presence of untreated hydrosalpinx; (5) transferred embryo receiving preimplantation genetic testing; and (6) transferred embryo derived from oocyte donation or oocyte cryopreservation. Each woman was included only once in the study.

### Outcome Definitions

The primary outcome was live birth after frozen embryo transfer (FET), which was defined as the delivery of any neonate with signs of life at 24 weeks of gestation or later. Secondary outcomes were as follows. Conception was defined as a positive pregnancy test result with a serum human chorionic gonadotropin level of 10 IU/L or greater 12 days after FET. Clinical pregnancy was defined as ultrasound confirmation of at least 1 intrauterine gestational sac 30 to 35 days after FET. Biochemical pregnancy loss was defined as a positive pregnancy test result followed by a decrease in human chorionic gonadotropin level, without any ultrasonographic evidence of intrauterine or extrauterine pregnancy. Clinical pregnancy loss was defined as the loss of a clinically recognized intrauterine pregnancy before 24 weeks of gestation.^11^ Total pregnancy loss consisted of biochemical pregnancy loss and CPL. Preterm birth was defined as a birth that occurred before 37 weeks of gestation. Low birth weight (LBW) referred to neonates born weighing less than 2500 g. Small for gestational age and large for gestational age were defined as birthweight below the 10th percentile and above the 90th percentile for gestational age based on a sex-specific reference,^12^ respectively. A healthy live birth was defined as a singleton live birth delivered at 37 weeks of gestation or later, with birth weight between the 10th and 90th percentiles. Details of the calculation methods for these outcomes are provided in the eTable in Supplement 1.

### Statistical Analysis

Data are expressed as means (SDs) for normally distributed continuous variables, medians (IQRs) for nonnormally distributed continuous variables, and frequencies (percentages) for categorical variables. Comparisons among different groups were performed using 1-way analysis of variance or the Kruskal-Wallis test for continuous variables and the χ^2^ or Fisher exact test for categorical variables.

The IPI was determined by calculating the length of the time interval between the day of the preceding CPL and the first day of the menstrual cycle during which the endometrial preparation for FET was performed. Interpregnancy interval length was classified as less than 3 months, 3 to less than 6 months, and 6 to 12 months. Odds ratios (ORs) were estimated with multivariable logistic regression analysis to evaluate the association between IPI and pregnancy outcomes, with individuals with an IPI of 6 to 12 months set as the reference group. Variables that differed across the groups with a *P* < .10 and those of clinical relevance were included in the multivariable model. These variables included maternal age (continuous), body mass index (continuous [calculated as weight in kilograms divided by height in meters squared]), parity, diagnosis of polycystic ovary syndrome, anti-Mullerian hormone level (continuous), trimester stage of the preceding CPL (first trimester vs second trimester), means of pregnancy termination for the preceding CPL (surgical vs nonsurgical evacuation), fertilization methods (IVF vs intracytoplasmic sperm injection), endometrial thickness before FET (continuous), endometrial preparation methods used for FET (natural regimen vs programmed regimen vs stimulated regimen), number of the transferred embryo (1 vs 2), developmental stage of the transferred embryo (D5 vs D6 or D7), and blastocyst scores of the transferred embryo^13^ (AA + AB + BA vs BB vs others). To evaluate whether there was a dose-response association between IPI and pregnancy outcomes, a test for linear trend was performed by modeling the median values of each category of IPIs as a continuous variable. We also conducted stratified analyses and interaction analyses for the primary outcome (live birth) to examine whether the association between IPI and the probability of achieving live birth differed by several potential confounding factors. Interaction between categorical IPIs and any of the stratifying variables (dichotomous) was evaluated by entering a multiplicative interaction term into the model. The *P* value for interaction was determined via a likelihood ratio test comparing models with and without the interaction terms.

Statistical analyses were performed using R software, version 4.0.3 (R Project for Statistical Computing); *P* < .05 (2-sided) indicated statistical significance. Data analysis was performed from April to May 2023.

## Results

This study included 2433 women (mean [SD] age, 31.8 [4.6] years) who received IVF treatment (Figure). There were 338 women (13.9%) with an IPI of less than 3 months, 1347 (55.4%) with an IPI of 3 to less than 6 months, and 748 (30.7%) with an IPI of 6 to 12 months. Table 1 summarizes the baseline characteristics. The median (IQR) IPI for the 3 groups was 77 (65-85) days, 128 (109-152) days, and 234 (202-288) days, respectively.

**Figure.**
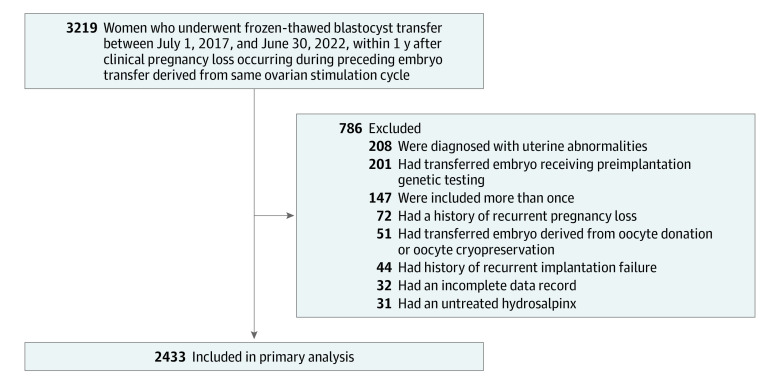
Flowchart of the Study Population

**Table 1.** Distribution of Baseline Demographics and Cycle Characteristics According to Categorical IPIs

Characteristic	IPI^a^			*P* value
	<3 mo (n = 338)	3 to <6 mo (n = 1347)	6-12 mo (n = 748)	
IPI length, median (IQR), d	77 (65-85)	128 (109-152)	234 (202-288)	<.001
Maternal age, y				
Mean (SD)	32.4 (4.8)	31.7 (4.6)	31.8 (4.5)	.05
≥35	114 (33.7)	380 (28.2)	195 (26.1)	.03
Paternal age, mean (SD), y	33.8 (5.2)	33.6 (5.1)	33.7 (5.2)	.68
BMI, mean (SD)	23.9 (3.5)	24.4 (3.7)	24.5 (3.8)	.05
Parity				
0	189 (55.9)	872 (64.7)	523 (69.9)	<.001
1	128 (37.9)	428 (31.8)	202 (27.0)	
≥2	21 (6.2)	47 (3.5)	23 (3.1)	
Previous abortions				
1	236 (69.8)	943 (70.0)	510 (68.2)	.68
2	67 (19.8)	261 (19.4)	165 (22.1)	
≥3	35 (10.4)	143 (10.6)	73 (9.8)	
Cause of infertility				
Tubal factors	250 (74.0)	955 (70.9)	524 (70.1)	.48
Male factors	57 (16.9)	235 (17.4)	127 (17.0)	
Others	31 (9.2)	157 (11.7)	97 (13.0)	
Diagnosis of PCOS	47 (13.9)	233 (17.3)	164 (21.9)	.003
AMH level, ng/mL				
Median (IQR)	3.0 (1.8-5.2)	3.8 (2.3-6. 6)	3.8 (2.0- 6.3)	<.001
<1.2	41 (12.1)	99 (7.3)	82 (11.0)	.003
No. of retrieved oocytes, mean (SD)	12.9 (6.2)	13.3 (6.3)	13.1 (6.8)	.55
Fertilization method				
IVF	260 (76.9)	938 (69.6)	522 (69.8)	.02
ICSI	78 (23.1)	409 (30.4)	226 (30.2)	
Endometrial preparation method for FET				
Natural regimen	216 (63.9)	691 (51.3)	305 (40.8)	<.001
Programmed regimen	96 (28.4)	535 (39.7)	348 (46.5)	
Stimulated regimen	26 (7.7)	121 (9.0)	95 (12.7)	
EMT before FET, mean (SD), cm	0.99 (0.2)	0.98 (0.2)	0.95 (0.2)	<.001
EMT <8 mm	25 (7.4)	106 (7.9)	89 (11.9)	.005
No. of transferred embryos				
1	322 (95.3)	1282 (95.2)	720 (96.3)	.50
2	16 (4.7)	65 (4.8)	28 (3.7)	
Developmental stage of transferred embryo				
D5	250 (74.0)	1021 (75.8)	550 (73.5)	.48
D6 or D7	88 (26.0)	326 (24.2)	198 (26.5)	
Blastocyst scores of transferred embryo				
AA + AB + BA	115 (34.0)	420 (31.2)	209 (27.9)	.13
BB	186 (55.0)	734 (54.5)	422 (56.4)	
Other	37 (10.9)	193 (14.3)	117 (15.6)	
Type of embryo transfer in cycle of preceding CPL				
Fresh embryo transfer	163 (48.2)	603 (44.8)	336 (44.9)	.51
FET	175 (51.8)	744 (55.2)	412 (55.1)	
Gestational age of preceding CPL, median (IQR), d	61 (56-66)	64 (59-77)	67 (59-110)	<.001
Trimester stage of preceding CPL				
First	313 (92.6)	1059 (78.6)	486 (65.0)	<.001
Second	25 (7.4)	288 (21.4)	262 (35.0)	
Surgical termination for the preceding CPL	124 (36.7)	574 (42.6)	340 (45.5)	.03

Abbreviations: AMH, anti-Mullerian hormone; BMI, body mass index (calculated as weight in kilograms divided by height in meters squared); CPL, clinical pregnancy loss; EMT, endometrial thickness; FET, frozen embryo transfer; ICSI, intracytoplasmic sperm injection; IPI, interpregnancy interval; IVF, in vitro fertilization; PCOS, polycystic ovary syndrome.

^a^ Unless indicated otherwise, values are presented as No. (%) of patients.

Women with a short IPI (<3 months) vs those with a longer IPI (3 to <6 or 6-12 months) were more likely to be older (aged ≥35 years; 114 [33.7%] vs 380 [28.2%] or 195 [26.1%]) and less likely to be nulliparous (189 [55.9%] vs 872 [64.7%] or 523 [69.9%]). Fewer women with shorter IPIs (<3 or 3 to <6 months) vs those with a longer IPI (6-12 months) were diagnosed with polycystic ovarian syndrome (47 [13.9%] or 233 [17.3%] vs 164 [21.9%]), had an endometrial thickness of less than 8 mm before FET (25 [7.4%] or 106 [7.9%] vs 89 [11.9%]), and received a programmed regimen to prepare the endometrium for FET (96 [28.4%] or 535 [39.7%] vs 348 [46.5%]). Women with a short IPI (<3 months) vs those with a longer IPI (3 to <6 or 6-12 months) were less likely to experience a preceding CPL in the second trimester (25 [7.4%] vs 288 [21.4%] or 262 [35.0%]) and were less likely to undergo surgical evacuation of a preceding pregnancy (124 [36.7%] vs 574 [42.6%] or 340 [45.5%]).

Comparisons of pregnancy outcomes and perinatal complications among the 3 groups are shown in Table 2. Statistically significant differences were observed across the 3 groups in rates of live birth, singleton live birth, total pregnancy loss, and biochemical pregnancy loss. Rates of live birth (134 [39.6%] for an IPI of <3 months, 569 [42.2%] for an IPI of 3 to <6 months, and 353 [47.2%] for an IPI of 6-12 months) and singleton live birth (127 [37.6%], 547 [40.6%], and 346 [46.3%]) showed a decreased trend as the IPI shortened; the incidence of total pregnancy loss (85 of 218 [39.0%], 279 of 856 [32.6%], and 146 of 500 [29.2%]) presented the opposite tendency.

**Table 2.** Frequencies of Pregnancy Outcomes and Perinatal Outcomes According to Categorical IPIs

Outcome	IPI, No. (%) of patients			*P* value
	<3 mo (n = 338)	3 to <6 mo (n = 1347)	6-12 mo (n = 748)	
Live birth	134 (39.6)	569 (42.2)	353 (47.2)	.03
Singleton	127 (37.6)	547 (40.6)	346 (46.3)	.009
Twin	7 (2.1)	22 (1.6)	7 (0.9)	.28
Conception	218 (64.5)	856 (63.5)	500 (66.8)	.32
Clinical pregnancy	176 (52.1)	737 (54.7)	437 (58.4)	.10
Total pregnancy loss	85/218 (39.0)	279/856 (32.6)	146/500 (29.2)	.04
Biochemical pregnancy loss	42/218 (19.3)	111/856 (13.0)	62/500 (12.4)	.03
Clinical pregnancy loss	42/176 (23.9)	168/737 (22.8)	84/437 (19.2)	.28
In first trimester	31/176 (17.6)	122/737 (16.6)	55/437 (12.6)	.13
In second trimester	11/176 (6.2)	46/737 (6.2)	29/437 (6.6)	.96
Preterm birth	21/134 (15.7)	77/569 (13.5)	54/353 (15.3)	.69
LGA in singletons	27/127 (21.3)	133/547 (24.3)	78/346 (22.5)	.70
SGA in singletons	8/127 (6.3)	12/547 (2.2)	10/346 (2.9)	.06
LBW in singletons	9/127 (7.1)	30/547 (5.5)	32/346 (9.2)	.10
Healthy live birth	81 (24.0)	356 (26.4)	217 (29.0)	.19

Abbreviations: IPI, interpregnancy interval; LBW, low birth weight; LGA, large for gestational age; SGA, small for gestational age.

The results of logistic regression analyses showed that compared with an IPI of 6 to 12 months, shorter IPIs (<3 and 3 to <6 months) were associated with decreased odds of clinical pregnancy (AOR, 0.70 [95% CI, 0.53-0.92] and 0.79 [0.65-0.95]; *P* = .003 for trend), total live birth (AOR, 0.64 [95% CI, 0.48-0.85] and 0.74 [0.61-0.90]; *P* < .001 for trend), singleton live birth (AOR, 0.62 [95% CI, 0.47-0.81] and 0.73 [0.60-0.88]; *P* < .001 for trend), and healthy live birth (AOR, 0.63 [95% CI, 0.46-0.87] and 0.79 [0.64-0.98]; *P* = .003 for trend) (Table 3). Compared with women with an IPI of 6 to 12 months, women with shorter IPIs (<3 and 3 to <6 months) had a higher risk of total pregnancy loss (AOR, 1.87 [95% CI, 1.31-2.67] and 1.29 [1.00-1.66]; *P* = .002 for trend). Women with an IPI of less than 3 months had a higher risk of biochemical pregnancy loss (AOR, 1.95 [95% CI, 1.24-3.09]) than those with an IPI of 6 to 12 months.

**Table 3.** Crude and Adjusted Odds Ratios of Pregnancy Outcomes and Perinatal Outcomes According to Categorical IPIs

Outcome	IPI					*P* value for trend
	<3 mo		3 to <6 mo		6-12 mo	
	OR (95% CI)	*P* value	OR (95% CI)	*P* value	OR (95% CI)	
Total live birth						
Crude	0.74 (0.57-0.95)	.02	0.82 (0.68-0.98)	.03	1 [Reference]	.008
Adjusted^a^	0.64 (0.48-0.85)	.002	0.74 (0.61-0.90)	.002	1 [Reference]	<.001
Singleton live birth						
Crude	0.70 (0.54-0.91)	.008	0.79 (0.66-0.95)	.01	1 [Reference]	.002
Adjusted	0.62 (0.47-0.81)	.001	0.73 (0.60-0.88)	.001	1 [Reference]	<.001
Twin live birth						
Crude	2.24 (0.78-6.43)	.14	1.76 (0.75-4.13)	.20	1 [Reference]	.12
Adjusted	2.12 (0.65-6.92)	.22	1.71 (0.69-4.27)	.25	1 [Reference]	.18
Conception						
Crude	0.90 (0.69-1.18)	.45	0.86 (0.72-1.04)	.13	1 [Reference]	.19
Adjusted	0.83 (0.62-1.10)	.20	0.79 (0.65-0.97)	.02	1 [Reference]	.04
Clinical pregnancy						
Crude	0.77 (0.60-1.00)	.05	0.86 (0.72-1.03)	.10	1 [Reference]	.04
Adjusted	0.70 (0.53-0.92)	.01	0.79 (0.65-0.95)	.01	1 [Reference]	.003
Total pregnancy loss						
Crude	1.55 (1.11-2.16)	.01	1.17 (0.92-1.49)	.19	1 [Reference]	.02
Adjusted	1.87 (1.31-2.67)	.001	1.29 (1.00-1.66)	.046	1 [Reference]	.002
Biochemical pregnancy loss						
Crude	1.69 (1.10-2.59)	.02	1.05 (0.75-1.47)	.76	1 [Reference]	.10
Adjusted	1.95 (1.24-3.09)	.004	1.16 (0.82-1.64)	.40	1 [Reference]	.03
Clinical pregnancy loss						
Crude	1.32 (0.87-2.01)	.20	1.24 (0.93-1.66)	.15	1 [Reference]	.11
Adjusted	1.56 (0.99-2.45)	.05	1.35 (0.99-1.83)	.06	1 [Reference]	.03
Preterm birth						
Crude	1.03 (0.59-1.78)	.92	0.87 (0.59-1.26)	.46	1 [Reference]	.70
Adjusted	1.18 (0.65-2.16)	.59	0.91 (0.61-1.36)	.65	1 [Reference]	.98
LGA in singletons						
Crude	0.93 (0.57-1.52)	.77	1.10 (0.80-1.52)	.54	1 [Reference]	.84
Adjusted	1.12 (0.66-1.91)	.68	1.22 (0.87-1.71)	.26	1 [Reference]	.35
SGA in singletons						
Crude	2.26 (0.87-5.86)	.09	0.75 (0.32-1.76)	.51	1 [Reference]	.44
Adjusted	2.33 (0.78-6.97)	.13	0.61 (0.24-1.52)	.29	1 [Reference]	.68
LBW in singletons						
Crude	0.75 (0.35-1.62)	.46	0.57 (0.34-0.96)	.03	1 [Reference]	.07
Adjusted	0.86 (0.37-1.97)	.72	0.58 (0.33-1.00)	.048	1 [Reference]	.14
Healthy live birth						
Crude	0.77 (0.57-1.04)	.09	0.88 (0.72-1.07)	.20	1 [Reference]	.08
Adjusted	0.63 (0.46-0.87)	.004	0.79 (0.64-0.98)	.03	1 [Reference]	.003

Abbreviations: IPI, interpregnancy interval; LBW, low birth weight; LGA, large for gestational age; OR, odds ratio; SGA, small for gestational age.

^a^ Adjusted for maternal age, body mass index, parity, diagnosis of polycystic ovary syndrome, anti-Mullerian hormone level, trimester stage of the preceding clinical pregnancy loss, means of pregnancy termination for the preceding clinical pregnancy loss, fertilization methods, endometrial thickness before frozen embryo transfer, endometrial preparation methods used for frozen embryo transfer, the number of the transferred embryo, developmental stage of the transferred embryo, and blastocyst scores of the transferred embryo.

Subgroup analyses were performed with regard to live birth stratified according to maternal age, endometrial thickness before FET, trimester stage of the preceding CPL, and means of pregnancy termination for the preceding CPL (eFigure in Supplement 1). However, no statistically significant interactions were observed between categorical IPIs and any of the relevant subgroups. Generally, the trend of a decreased probability of achieving live birth as the IPI shortened was consistent across subgroups stratified by the confounding factors.

## Discussion

Compared with an IPI of 6 to 12 months between a preceding CPL and a subsequent FET, we observed that shorter IPIs of less than 3 months or 3 to less than 6 months were associated with a decreased likelihood of achieving clinical pregnancy, live birth, and healthy live birth and an increased risk of total pregnancy loss after FET.

Whether to delay a pregnancy attempt after a CPL remains controversial. The World Health Organization recommends a minimum IPI of 6 months after a spontaneous or induced abortion based on higher risks of LBW, preterm birth, and premature rupture of membranes with a shorter IPI (<6 months) as reported in 2005 in a retrospective study conducted in Latin America.^6^ However, subsequent studies in naturally conceiving populations suggest that delaying an attempt of the next pregnancy for at least 6 months may be unnecessary.^7,14,15^ A 2017 meta-analysis^7^ pooled results from 10 studies and indicated that compared with an IPI of 6 months or longer, an IPI of less than 6 months after miscarriage was not associated with an increased risk of adverse outcomes, including stillbirth, preeclampsia, and LBW; rather, it was associated with favorable outcomes in terms of a lower risk of reexperiencing miscarriage and a greater likelihood of achieving live birth in the next pregnancy. There are several possible reasons for the divergent results between these studies with naturally conceived pregnancies and our study with IVF treatment. In most of the previous studies^15,16,17^ in naturally conceiving populations, IPI length was generally defined as the period between the end of the preceding CPL and the onset of the last menstrual cycle of the next pregnancy, which was likely confounded by female fecundity. That is, women who are able to conceive naturally sooner after a preceding CPL (ie, shorter IPI) may have better fecundity, whereas women with a longer IPI are more likely to experience compromised fecundity or subfertility, resulting in a lower likelihood of achieving live birth that possibly counteracts the benefit of longer IPIs on pregnancy outcomes.^18^ Although the results remained unaltered when taking into account the duration of trying to conceive,^14,19^ there may have been a possibility of self-reporting bias or recall bias (eg, the estimated date of the last menstrual period or the recall of the duration couples spent trying to conceive). In addition, we observed a higher risk of biochemical pregnancy loss among women with an IPI of less than 3 months compared with those with an IPI of 6 to 12 months. However, the outcome of biochemical pregnancy loss was rarely reported in the previous studies involving naturally conceived pregnancies that generally referred to an ultrasound confirmation of the intrauterine gestational sac or sacs.

Evidence regarding IPI after CPL in infertile women is limited. Among 257 women undergoing IVF treatment, Sharon-Weiner et al^10^ found that the time interval between a preceding pregnancy loss in the first trimester and the subsequent IVF cycle had no association with the chance of achieving a clinical pregnancy. It is noteworthy that the investigators included a preceding total pregnancy loss consisting of both a CPL and a biochemical pregnancy loss, but the latter may commonly require faster recovery time before initiation of the next embryo transfer. Moreover, the relatively small sample size may have limited the power to detect a statistically significant difference in terms of pregnancy or live birth.

Our results suggest that an IPI allowing at least 6 months for recovery may benefit the subsequent FET for women who experience a CPL during the preceding embryo transfer, in terms of an increased probability of achieving clinical pregnancy and live birth. The causal mechanisms that underlie the association between a short IPI and unfavorable pregnancy outcomes remain unclear. It seems that shorter IPIs may be associated with inadequate time for recovery from the physiologic and psychological stresses resulting from the preceding CPL. On one hand, endometrial damage (eg, bleeding, infection, adhesion), cervical dilatation, or alterations in the uterine microbiome caused by surgical procedures perhaps call for allowing an adequate period to fully return to physiologic status.^20^ On the other hand, psychological factors such as depressive symptoms, anxiety, and distress have been reported to reduce the chances of becoming pregnant.^21,22^ Infertile women undergoing IVF treatment are commonly confronted with a higher level of psychological and emotional stress compared with women who conceive naturally.^23,24^ Women experiencing a CPL after embryo transfer may be more vulnerable to psychological morbidity, including anxiety, depression, and posttraumatic stress,^25,26,27^ thereby likely taking longer to achieve complete recovery.

Consistent with previous studies involving natural conception, we did not observe associations of IPI after a preceding CPL with the risk of preterm birth, LBW, and SGA after the next embryo transfer. The “depletion hypothesis” has been proposed to partially explain the association between a short IPI after delivery and unfavorable perinatal outcomes.^4,28^ This hypothesis speculates that the depletion of several maternal nutrients that occur during the preceding pregnancy and during lactation cannot recover to prepregnancy physiologic status in a short period, resulting in a suboptimal environment for fetal growth and, consequently, an increased risk of adverse perinatal outcomes. The results of our study and previous studies involving natural conception suggest that women who have experienced a preceding CPL may not experience maternal nutritional depletion that was possibly related to an increased risk of perinatal complications.

The association between the surgical evacuation of pregnancy and the pregnancy outcomes of subsequent embryo transfer remains debatable. Ozgur et al^20^ suggested that women who underwent surgical management for CPL may require 6 months for endometrial functional recovery to optimize the chance of achieving a live birth after FET. A previous study reported that there was no notable difference in pregnancy outcomes of subsequent embryo transfer for women who underwent surgical evacuation compared with those who did not.^29^ Surgical evacuation may lead to endometrial damage because of potential complications such as infection and intrauterine adhesion, and thus may play a causal role in endometrial thinning that is closely associated with unfavorable pregnancy outcomes.^30,31^ In our study, women with an IPI of 6 to 12 months were more likely to undergo surgical evacuation of the preceding CPL and were more likely to have an endometrial thickness of less than 8 mm before FET compared with those with shorter IPIs. However, we did not observe a notable interaction between the mean of pregnancy termination for the preceding CPL and categorical IPIs in terms of live birth. Although a positive association of decreased probability of achieving live birth with shortened IPIs was observed only among women receiving nonsurgical evacuation, the subgroup analyses might be underpowered to detect the interaction because of the limited sample size.

### Limitations

This study was limited by its single-center and retrospective nature. Despite adjusting for multiple potential confounders, the possibility of selection bias and confounding bias by residual confounding or confounding by indication could not be ruled out. Particularly, women with advanced age or decreased ovarian reserve commonly manifested a shorter menstrual cycle, which was likely a contributor to shortened time intervals between a preceding CPL and the next pregnancy attempt. Nevertheless, advanced age or declined ovarian reserve also may be indicative of poor prognosis. In addition, the number of previous embryo transfer cycles may be a potential unmeasured confounder even though women with recurrent implantation failure were excluded from the study. Finally, considering that few women would wait for more than 1 year in the case of remaining embryos, only women who underwent FET within 1 year after a preceding CPL were included in this study. Thus, these results may not be applicable to women with an IPI longer than 1 year. Despite these limitations, IVF treatment is one possible way to assess the association between IPI and subsequent pregnancy outcomes with less confounding from fecundity. In addition, fresh embryo transfer was excluded in our study because of the possible adverse effect of controlled ovarian stimulation on pregnancy outcomes. Furthermore, the recording of treatments and outcomes in our study was obtained from electronic medical records based on well-conducted follow-up visits, with a lower risk of recall bias than that in naturally conceived pregnancies.

## Conclusions

The findings of this cohort study suggest that delaying FET for at least 6 months after a preceding CPL was associated with beneficial pregnancy outcomes, considering that a decreased likelihood of achieving clinical pregnancy and live birth and an increased risk of total pregnancy loss was observed among women with shorter IPIs. Further prospective studies are needed to confirm our findings.

## Notes

Supplement 1. **eTable.** Details of the Calculation Methods for Pregnancy Outcomes **eFigure.** Stratified Analyses of the Association Between Categorical Interpregnancy Intervals (IPIs) and Live Births

Supplement 2.
Data Sharing Statement
