# Supplementary material for: Interpregnancy Interval After Clinical Pregnancy Loss and Outcomes of the Next Frozen Embryo Transfer
Source: JAMA Netw Open. 2023 Oct 31;6(10):e2340709. doi: 10.1001/jamanetworkopen.2023.40709 (PMC10618845; doi:10.1001/jamanetworkopen.2023.40709)
Supplement: Supplement 1. — eTable. Details of the Calculation Methods for Pregnancy Outcomes eFigure. Stratified Analyses of the Association Between Categorical Interpregnancy Intervals (IPIs) and Live Births [file jamanetwopen-e2340709-s001.pdf]

Supplementary Online Content

Wang Z, Meng Y, Shang X, et al. Interpregnancy interval after clinical pregnancy loss and outcomes of the next frozen embryo transfer. *JAMA Netw Open*. 2023;6(11):e2340709. doi:10.1001/jamanetworkopen.2023.40709

eTable. Details of the Calculation Methods for Pregnancy Outcomes

eFigure. Stratified Analyses of the Association Between Categorical Interpregnancy Intervals (IPIs) and Live Births

This supplementary material has been provided by the authors to give readers additional information about their work.

© 2023 Wang Z et al. *JAMA Network Open*.

eTable. Details of the Calculation Methods for Pregnancy Outcomes

Outcomes	Calculation methods
**Live birth**	calculated by dividing the number of women achieving live birth after FET (numerator) by the number of women receiving FET (denominator).
**Singleton live birth**	calculated by dividing the number of women achieving singleton live birth after FET (numerator) by the number of women receiving FET (denominator).
**Twin live birth**	calculated by dividing the number of women achieving twin live birth after FET (numerator) by the number of women receiving FET (denominator).
**Conception**	calculated by dividing the number of women having a positive pregnancy test with the serum human chorionic gonadotropin level of ≥10 IU/L 12 days after FET (numerator) by the number of women receiving FET (denominator).
**Clinical pregnancy**	calculated by dividing the number of women achieving a clinical pregnancy after FET (numerator) by the number of women receiving FET (denominator).
**Total pregnancy loss**	calculated by dividing the number of women experiencing both biochemical pregnancy loss and clinical pregnancy loss after FET (numerator) by the number of women having a positive pregnancy test after FET (denominator).
**Biochemical pregnancy loss**	calculated by dividing the number of women experiencing a biochemical pregnancy loss after FET (numerator) by the number of women having a positive pregnancy test after FET (denominator).
**Clinical pregnancy loss**	calculated by dividing the number of women experiencing a clinical pregnancy loss after FET (numerator) by the number of women achieving a clinical pregnancy after FET (denominator).
**Preterm birth**	calculated by dividing the number of women who given birth preterm after FET (numerator) by the number of women achieving live birth after FET (denominator).
**LGA in singletons**	calculated by dividing the number of women who delivered a singleton infant with the birthweight diagnosed with LGA after FET (numerator) by the number of women achieving singleton live birth after FET (denominator).
**SGA in singletons**	calculated by dividing the number of women who delivered a singleton infant with the birthweight diagnosed with SGA after FET (numerator) by the number of women achieving singleton live birth after FET (denominator).
**LBW in singletons**	calculated by dividing the number of women who delivered a singleton infant with the birthweight diagnosed with LBW after FET (numerator) by the number of women achieving singleton live birth

© 2023 Wang Z et al. *JAMA Network Open*.

	after FET (denominator).
**Healthy live birth**	calculated by dividing the number of women achieving a healthy live birth after FET (numerator) by the number of women receiving FET (denominator).

Abbreviations: FET, frozen embryo transfer; IPI, interpregnancy interval; LGA, large for gestational age; SGA, small for gestational age; LBW, low birth weight.

© 2023 Wang Z et al. *JAMA Network Open*.

eFigure. Stratified Analyses of the Association Between Categorical Interpregnancy Intervals (IPIs) and Live Births

Stratified Factors	aOR (95%CI)	*P* for interaction
**Age <35**		**0.74**
IPI of 6–12 months	1[Reference]	
IPI of 3 to <6 months	0.71(0.57–0.89)	
IPI of <3 months	0.62(0.45–0.87)	
**Age ⩾35**		
IPI of 6–12 months	1[Reference]	
IPI of 3 to <6 months	0.80(0.55–1.17)	
IPI of <3 months	0.74(0.43–1.25)	
**Preceding CPL in the first trimester**		**0.47**
IPI of 6–12 months	1[Reference]	
IPI of 3 to <6 months	0.74(0.59–0.92)	
IPI of <3 months	0.60(0.44–0.81)	
**Preceding CPL in the second trimester**		
IPI of 6–12 months	1[Reference]	
IPI of 3 to <6 months	0.75(0.53–1.08)	
IPI of <3 months	1.07(0.44–2.56)	
**Non–surgical evacuation of the preceding CPL**		**0.83**
IPI of 6–12 months	1[Reference]	
IPI of 3 to <6 months	0.69(0.54–0.90)	
IPI of <3 months	0.57(0.40–0.82)	
**Surgical evacuation of the preceding CPL**		
IPI of 6–12 months	1[Reference]	
IPI of 3 to <6 months	0.79(0.59–1.04)	
IPI of <3 months	0.73(0.46–1.13)	
**EMT ⩾ 8mm before FET**		**0.86**
IPI of 6–12 months	1[Reference]	
IPI of 3 to <6 months	0.75(0.61–0.91)	
IPI of <3 months	0.64(0.47–0.85)	
**EMT < 8mm before FET**		
IPI of 6–12 months	1[Reference]	
IPI of 3 to <6 months	0.59(0.30–1.14)	
IPI of <3 months	0.54(0.18–1.54)	

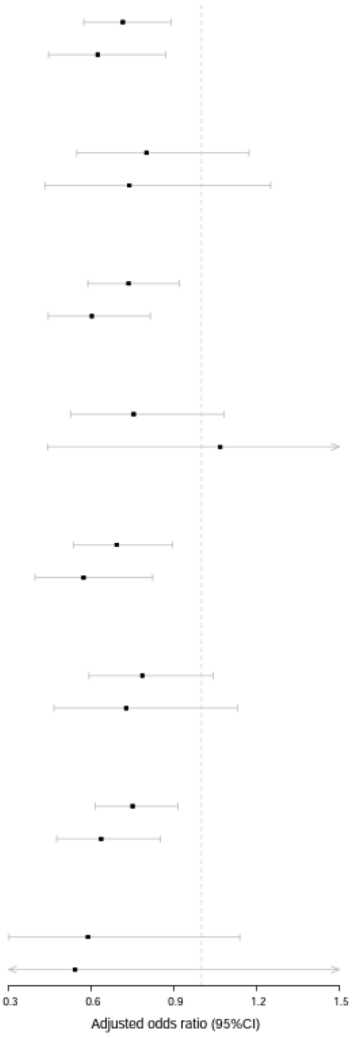

© 2023 Wang Z et al. *JAMA Network Open*.
