# Supplementary material for: Interpregnancy Interval After Clinical Pregnancy Loss and Outcomes of the Next Frozen Embryo Transfer
Source: JAMA Netw Open. 2023 Oct 31;6(10):e2340709. doi: 10.1001/jamanetworkopen.2023.40709 (PMC10618845; doi:10.1001/jamanetworkopen.2023.40709)
Supplement: Supplement 2. — Data Sharing Statement [file jamanetwopen-e2340709-s002.pdf]

Data Sharing Statement

Wang. Interpregnancy Interval After Clinical Pregnancy Loss and Outcomes of the Next Frozen Embryo Transfer. *JAMA Netw Open*. Published October 31, 2023.
doi:10.1001/jamanetworkopen.2023.40709

Data
Data available: Yes
Data types: Deidentified participant data
How to access data: The data of this study are available on request from the corresponding author (sdweidaimin@163.com)
When available: With publication

Supporting Documents
Document types: None

Additional Information
Who can access the data: The data will be made available for researchers whose proposed use of the data has been approved.
Types of analyses: The types of analyses for which the data will be made available for a specified purpose.
Mechanisms of data availability: The data will be made available after approval of a proposal.
